# Field evaluation of *TaDREB2B-*ectopic expression sugarcane (*Saccharum* spp. hybrid) for drought tolerance

**DOI:** 10.3389/fpls.2022.963377

**Published:** 2022-11-01

**Authors:** Shenghua Xiao, Yang Wu, Shiqiang Xu, Hongtao Jiang, Qin Hu, Wei Yao, Muqing Zhang

**Affiliations:** Guangxi Key Lab for Sugarcane Biology, State Key Lab for Conservation and Utilization of Subtropical Agri-Biological Resources, College of Agriculture, Guangxi University, Nanning, China

**Keywords:** sugarcane, *Prd29A: TaDREB2B*, stress-inducible promoter, drought tolerance, yield

## Abstract

Sugarcane is one of the most crucial sugar crops globally that supplies the main raw material for sugar and ethanol production, but drought stress causes a severe decline in sugarcane yield worldwide. Enhancing sugarcane drought resistance and reducing yield and quality losses is an ongoing challenge in sugarcane genetic improvement. Here, we introduced a *Tripidium arundinaceum* dehydration-responsive element-binding transcription factor (*TaDREB2B*) behind the drought-responsible *RD29A* promoter into a commercial sugarcane cultivar FN95-1702 and subsequently conducted a series of drought tolerance experiments and investigation of agronomic and quality traits. Physiological analysis indicated that *Prd29A: TaDREB2B* transgenic sugarcane significantly confers drought tolerance in both the greenhouses and the field by enhancing water retention capacity and reducing membrane damage without compromising growth. These transgenic plants exhibit obvious improvements in yield performance and various physiological traits under the limited-irrigation condition in the field, such as increasing 41.9% yield and 44.4% the number of ratooning sugarcane seedlings. Moreover, *Prd29A: TaDREB2B* transgenic plants do not penalize major quality traits, including sucrose content, gravity purity, Brix, etc. Collectively, our results demonstrated that the *Prd29A-TaDREB2B* promoter-transgene combination will be a useful biotechnological tool for the increase of drought tolerance and the minimum of yield losses in sugarcane.

## Introduction

Sugarcane (*Saccharum* spp. hybrid) acts as one of the important commercial crops that provides at least 60% of world sugar production (Rocha et al., 2007). In China, the main sugarcane area widely is distributed in the dry parts of several southern provinces, such as Guangxi and Yunnan (Que et al., 2012). Because of continuous rainfall shortages in recent years and large areas of sugarcane cultivation in the sloping field, water deficit has become one of the most important constraints in sugarcane growth, development, and biomass. Drought stress retards the expansion of sugarcane leaves, inhibits leaf photosynthesis, decreases the absorption capacity of nitrogen, and impacts other physiological processes, finally resulting in severe losses of sugar yield (Li et al., 2016; Garcia Tavares et al., 2018; Xu et al., 2018). It is necessary to create new sugarcane cultivars possessing excellent drought tolerance or water-saving characteristics to mitigate the negative effects resulting from the ongoing water shortage. Transgenic technologies have gradually become promising biotechnological tools for developing new stress-resistance crop varieties in recent years (Younis et al., 2014), but few reports on using transgenic technology in sugarcane breeding. So far, although tremendous efforts to develop drought-resistant sugarcane, few attempts have improved drought resistance without sacrificing yield under actual field conditions (Augustine et al., 2015b; Augustine et al., 2015a).

For over 50 years, sugarcane breeders introgress agronomically valuable traits from sugarcane relatives to enlarge the natural growing range of sugarcane, improve a series of biotic and abiotic stress tolerance, and enhance its resource efficiency (Wu et al., 2014; Augustine et al., 2015a; Yu et al., 2022). *Tripidium arundinaceum*, a relative wild sugarcane species, is grass with tall stalks, long internodes, and low sugar contents (Ram et al., 2001; Augustine et al., 2015b; Lloyd Evans et al., 2019). Besides outstanding vigor and high fiber content, *T. arundinaceum* has a strong perennial ratooning ability and can produce many tillers with a rapid growth rate (Ram et al., 2001). Moreover, it possesses excellent resistance to drought, waterlogging, barrenness, and diseases (Ram et al., 2001; Que et al., 2012). Under drought stress, *T. arundinaceum* shows significantly higher scavenging ability to active oxygen, osmotic adjusting ability, and photosynthetic rate than sugarcane (Que et al., 2012; Manoj et al., 2019). The experiments on drought and salt tolerance also reveal that *T. arundinaceum* displayed better tolerance than sugarcane in physical appearance (Que et al., 2012). Isolating and utilizing the drought-tolerance gene from *T. arundinaceum* might be effective for engineering drought-tolerant sugarcane cultivars. However, most of the studies on *T. arundinaceum* mainly focused on its morphology and chromosomal characterization (Wu et al., 2014; Huang et al., 2017; Yang et al., 2019; Yu et al., 2021; Yu et al., 2022).

Increasing essential genes regulating drought tolerance have been identified and characterized in plants. The DREB transcription factors are widely present in many plant species and show to enhance plant tolerance to various abiotic stresses such as drought stress (Kudo et al., 2017; Xie et al., 2019; Zhou et al., 2020; Yang et al., 2020b). In apples, overexpression of *MsDREB6.2* improves drought tolerance through accelerating root growth and reducing stomatal opening (Liao et al., 2017). The DREB2 protein in *Arabidopsis* is a well-known positive regulator of drought-responsive gene expression (Sakuma et al., 2006). *TINY* gene in *Arabidopsis* positively regulates plant drought tolerance through activating the expression of drought-responsive genes and promoting ABA-mediated stomatal closure (Xie et al., 2019). Increasing evidence reported that the DREB subfamily members increase the stress durability in plants through reprogramming downstream stress-responsive genes if overexpressed under the drive of solid constitutive promoters (Xianjun et al., 2011; Agarwal et al., 2017; Xie et al., 2019; Xiao et al., 2021). Unfortunately, constitutive overexpression of stress-related regulatory genes usually harmed growth and yield under normal growth conditions (Morran et al., 2011; Agarwal et al., 2017; Selvaraj et al., 2020). More and more promoters of stress-inducible functional genes such as *OsNAC6* (Nakashima et al., 2007; Selvaraj et al., 2020), *OsWRKY71* (Kovalchuk et al., 2013), *ZmRab17* (Morran et al., 2011), and *TdCor39* (Kovalchuk et al., 2013) and promoters of stress-inducible regulatory genes such as *LIP19* (Nakashima et al., 2007), have been applied to biotechnology breeding to decrease these negative effects. Therefore, utilizing stress-inducible promoters to optimize expression levels of transgenesis is crucial to improving plant tolerance without yield losses simultaneously (Agarwal et al., 2017).

The *responsive to desiccation 29A* (*RD29A*) from *Arabidopsis* is a drought-responsive gene, several studies showed that its promoter had been used for moderate stress-inducible transgene expression in plants (Kasuga et al., 1999; Kasuga, 2004; Mallikarjuna et al., 2011). In our previous study, the *green fluorescent protein* (*GFP*) gene controlled by *RD29A* promoter (*Prd29A*) was introduced into sugarcane callus and showed that significantly induced by PEG (Wu et al., 2008). Therefore, *Prd29A* was expected to serve as a candidate for the drought-inducible promoters for sugarcane molecular breeding. In this study, we identified a gene *TaDREB2B* from *T. arundinaceum*, which is a homolog of *AtDREB2* in *Arabidopsis*. We constructed a plant expression vector that expressed the *TaDREB2B* gene driven by drought-inducible *Prd29A* and introduced it into a commercial sugarcane cultivar FN95-1702 to evaluate its functions under actual field conditions. To our knowledge, it is the first report whereby a single stress regulatory gene from sugarcane wild relative species was introduced into sugarcane to improve its stress resistance in the field. We provided sufficient evidence that expression of *TaDREB2B* under control of the *Prd29A* confers drought tolerance, increases agronomic performance, and ensures yield in the field under drought stress.

## Materials and methods

### *Plant materials, growth conditions*, drought and PEG treatment

Wild type (*Saccharum* spp. *Hybrid* cv. FN95-1702), empty vector (EV) and *Prd29A:TaDREB2B* transgenic sugarcane lines generated from cv. FN95-1702 were planted in a sugarcane field in Fuzhou, Fujian province, China. For asexually propagated sugarcane, all materials used in this study come from the first generation of transgenic sugarcane. The stems with sugarcane buds were grown in the fertile soil under well-watered conditions for growth under 16 h/8 h light/dark illumination, 27°C, and 60%-70% air humidity condition. For drought treatment, soil water content and membrane permeability were monitored and represent different levels of water deficit (well-watered, mild drought stress, severe drought stress, and re-watered) in the four-leaf stage sugarcane seedlings (Xu et al., 2018). For PEG treatment, the fertile soil with sugarcane seedlings at the four-leaf stage was soaked with 20% PEG-4000 and sugarcane leaves were evenly spraied with same PEG solution. The leaves of six individual plants at the +1 position were harvested after drought treatment and PEG treatment at 0, 6, 9, 12 and 48 h, and stored at -80°C until further use.

### Phylogenetic analysis and plant transformation of TaDREB2B

*Arabidopsis*, rice, maize, and sugarcane AP2/ERF protein sequences were obtained through BLAST analysis at the website (https://blast.ncbi.nlm.nih.gov/Blast.cgi). The amino acid sequence alignments were generated by DNAMAN software. The phylogenetic tree was constructed using the Clustal X, MEGA 7 software, and iTOL website (https://itol.embl.de/).

*TaDREB2B* gene from *T. arundinaceum* was inserted in *Prd29A-hyg* digested with *PstI* and *SphI* restriction enzyme (NEB, USA) using ClonExpress Ultra One Step Cloning Kit (Vazyme, China) according to our previous method (Wu et al., 2008). The generated *Prd29A-TaDREB2B-hyg* expression vector was used to transform sugarcane (Wu et al., 2008).

### Total RNA extraction and RT-qPCR analysis

The total RNA of sugarcane leaves was extracted using RNA Extraction Kit (Tiangen Biotech, Beijing, China) for gene expression, and 3 μg of total RNA was used to generate first-strand cDNA using SuperScript III reverse transcriptase (Invitrogen, CA, USA) and was diluted 50-100 times with ddH_2_O. Reverse transcription-quantitative PCR (RT-qPCR) was performed on a 7500 Real-Time PCR system (ABI, CA, USA) in a 15 μL reaction volume (Xiao et al., 2021). The house-keeping genes *25S rRNA* was used as internal controls. The primers sequence used in this study are *25S rRNA* (F:5’-ATAACCGCATCAGGTCTCCAAG-3’; R:5’-CCTATTGGTGGGTGAACAATCC-3’), *TaDREB2B* (F:5’-ATGATGAAGCGGCTAAGGTT-3’; R:5’-AAGAACCGCCTTATCCTCAA-3’).

### Measurement of drought-relevant physiological indicators

To determine MDA content, the crushed sugarcane leaf sample of 0.1 g was suspended with 1 mLof 0.1% trichloroacetic acid. After centrifugation at 12000 g for 2 min, 0.5 mL supernatant and 1mL of 10% trichloroacetic acid containing 0.67% thiobarbituric acid were mixed, subsequentlyboiled for 0.5 h, and rapidly cooled with ice. The OD values were measured and recorded at 450,532, and 600 nm, respectively (Luo et al., 2020).

The extracts were filtered and analyzed according to the anthrone–sulfuric acid method for soluble sugar content. Briefly, the crushed sugarcane leaf sample of 0.1 g was mixed with 0.5 mL of ddH_2_O, 0.05 mL of anthrone reagent and 0.5 mL concentrated sulfuric acid, then boiled at 100°C for 1-2 min. After cooling with ice water, the OD values were measured at 630 nm (Xin et al., 2020).

For electrolyte leakage measurement, sugarcane leaves were harvested and immersed in ddH_2_O, then vacuumed leaves were used to measure the conductivity and recorded as E1. After heating at 100°C for 0.5 h and cooling to 21-25°C, the conductivity was measured and recorded as E2. The relative electrical conductivity was calculated as E1/E2 × 100% (Luo et al., 2020).

### Agroindustrial performance of TaDREB2B transgenic sugarcane in the field trials

The field evaluations were performed in the sugarcane field in Fusui, Guangxi province, China. The stalks of the control and transgenic sugarcane were cut into single bud sets and sterilized with 5% carbendazim for 1 d, then each single bud set was planted in the soil in a randomized block design. Transgenic sugarcane lines and control plants were planted in 3 plots, respectively, with 3 rows and 30 sugarcane buds in each row. Agronomic and quality traits of all sugarcane plants were evaluated according to the previously described method (Arencibia et al., 1999; Gilbert et al., 2009; Yao et al., 2017).

### Membership function analysis

The membership function method evaluated the drought tolerance of WT and *TaDREB2B*-transgenic sugarcane lines. According to the previous method, the formula was U(X_i_) = (X_i_−X_min_)/(X_max_−X_min_) when the indicator was positively correlated with drought tolerance, and the formula was U(X_i_) = 1 − (X_i_ − X_min_)/(X_max_ − X_min_) when the indicator was negatively correlated (Xiong et al., 2022). X_i_= ΣU(p_m_ × X_i_)/n was the overall evaluation value, in which X_i_ is the measured value of an index of each sugarcane line. X_min_ and X_max_ represent the minimum and maximum values of the index, respectively. p_m_ is the weight coefficient of the m-th principal component and n is the number of indicators.

### Statistical analysis

All differences were analyzed using Statistix 8 software. For comparing two variables, differences analyses was performed using a Student’s *t*-test. For comparing three or more variables, differences analyses was performed using ANOVA and Tukey HSD multiple comparisons test.

### Accession numbers

The *Arabidopsis* gene sequences related to this work can be found in The Arabidopsis Information Resource (https://www.Arabidopsis.org/) under the following accession numbers: AtORA47 (AT1G74930), AtDREB26 (AT1G21910), AtDEAR5 (AT4G06746), AtDEAR1 (AT3G50260), AtDEAR3 (AT2G23340), AtDEAR4 (AT4G36900), AtDDF1 (AT1G12610), AtCBF4 (AT5G51990), AtCBF2 (AT4G25470), AtCBF1 (AT4G25490), AtCBF3 (AT4G25480), AtHARDY (AT2G36450), AtERF38 (AT2G35700), AtTINY (AT5G25810), AtTINY2 (AT5G11590), AtTINY3 (AT4G32800), AtESE2 (AT2G25820), AtABI4 (AT2G40220), AtDREB2B (AT3G11020), AtDREB2C (AT2G40340), AtWIND4 (AT5G65130), AtWIND3 (AT1G36060), AtWIND1 (AT1G78080), AtRAP2.4 (AT1G22190), AtCRF4 (AT4G27950), AtRAP2.6L (AT5G13330), AtERF71 (AT2G47520), AtERF72 (AT3G16770), AtERF96 (AT5G43410), AtERF4 (AT3G15210), AtERF10 (AT1G03800). Other gene sequence information can be obtained from National Library of Medicine (https://www.ncbi.nlm.nih.gov/) under the following accession numbers: OsDREB2A (XP_025878770.1), OsDREB2B (NP_001389444.1), SsDREB2B (Sspon.001C0004630), ZmDREB2A (PWZ09406.1).

## Results

### Phylogenetic analysis of TaDREB2B

**Figure 1:**
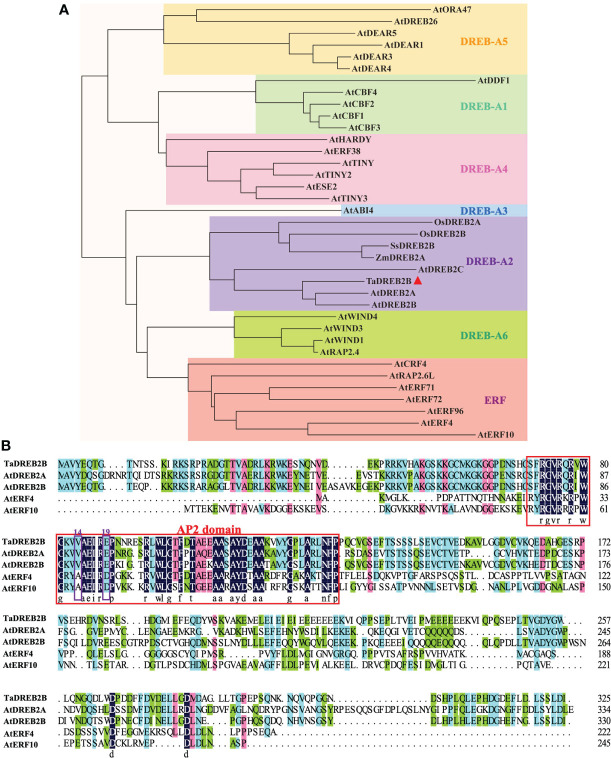
Phylogenetic tree and amino acid sequence alignment of TaDREB2B. **(A)** Phylogenetic analysis of TaDREB2B and other AP2/ERF transcription factors from *Arabidopsis*, rice, and maize. The red triangle represents the TaDREB2B protein. **(B)** Alignment of TaDREB2B, AtDREB2A, AtDREB2B, AtERF4 and AtERF10 protein sequences. The red rectangle represents the AP2 domain, and the purple rectangles show the amino acid on the 14th and 19th.

Based on the crucial role of DREB2 in plant drought response, we obtained a potential *TaDREB2B* gene from *T. arundinaceum* through BLAST analysis using the amino acid sequence of AtDREB2B. The *TaDREB2B* was cloned from *T. arundinaceum*, which contains a 978-bp open reading frame and encodes a protein with 325 amino acid residues. Phylogenetic tree analysis showed that TaDREB2B belongs to the DREB-A2 subfamily, and its orthologs are AtDREB2B (*Arabidopsis thaliana*), AtDREB2A, AtDREB2C, ZmDREB2A (*Zea mays* L.), SsDREB2B (*Saccharum spontaneum* L.) (**Figure 1A**). TaDREB2B protein contains an AP2/ERF domain with valine at 14th and glutamic acid at 19th (**Figure 1B**), suggesting that TaDREB2B is a typical member of the DREB subfamily transcription factor.

### The expression of TaDREB2B in transgenic sugarcane

**Figure 2:**
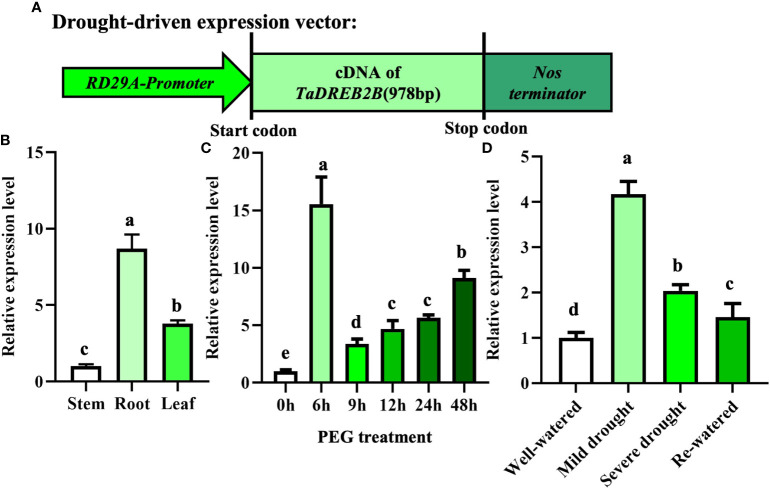
The expression of *TaDREB2B* in *Prd29A: TaDREB2B* transgenic sugarcane under water deficit conditions. **(A)** Schematic diagram of expression vector *Prd29A: TaDREB2B* used for sugarcane transformation. *RD29A-Promoter* represents the promoter of *the RD29A* gene in *Arabidopsis*. **(B)** The expression pattern of *TaDREB2B* in different tissues of transgenic sugarcane at the four-leaf stage after treatment with PEG. Samples of stem, root, and leaf were taken from plants at the four-leaf stage. The values are the means ± SD; n=3. **(C)** Expression of *TaDREB2B* at different time points following treatment of transgenic sugarcane seedlings at the four-leaf stage with PEG. The values are the means ± SD; n=3. **(D)** Relative expression of *TaDREB2B* following treatment of transgenic sugarcane seedlings subjected to different levels of water deficit. The values are the means ± SD; n=3. Different letters indicate significant differences as determined using ANOVA and LSD multiple comparisons (*P*<0.05).

Our previous studies generated a construct whereby the *TaDREB2B* gene was controlled by *RD29A* promoter (*Prd29A:TaDREB2B*) and introduced into sugarcane callus (**Figure 2A**), four *Prd29A:TaDREB2B* transgenic sugarcane lines subsequently were obtained (**Supplemental Figure 1**; Wu et al., 2008). To analyze whether *RD29A* promoter responds to drought stress, we first detected the transcript level of *TaDREB2B* in different tissues of *Prd29A:TaDREB2B* transgenic sugarcane after treatment with PEG by performing an RT-qPCR assay. *TaDREB2B* was ubiquitously expressed in all tissues with higher transcript levels in the root (**Figure 2B**). The expression level of *TaDREB2B* rapidly and markedly increased 15-fold at 6 h and merely increased 3~8 folds at 9-48 h in leaves of transgenic seedlings treated with PEG (**Figure 2C**). The expression pattern of *TaDREB2B* was further analyzed in sugarcane leaves subjected to different levels of water deficit. As shown in **Figure 2D**, the relative expression of *TaDREB2B* was significantly increased 4-fold under mild drought conditions while subsequently reduced after rehydration treatment (**Figure 2D**).

### Prd29A:TaDREB2B sugarcane confers drought tolerance without negatively affecting growth

**Figure 3:**
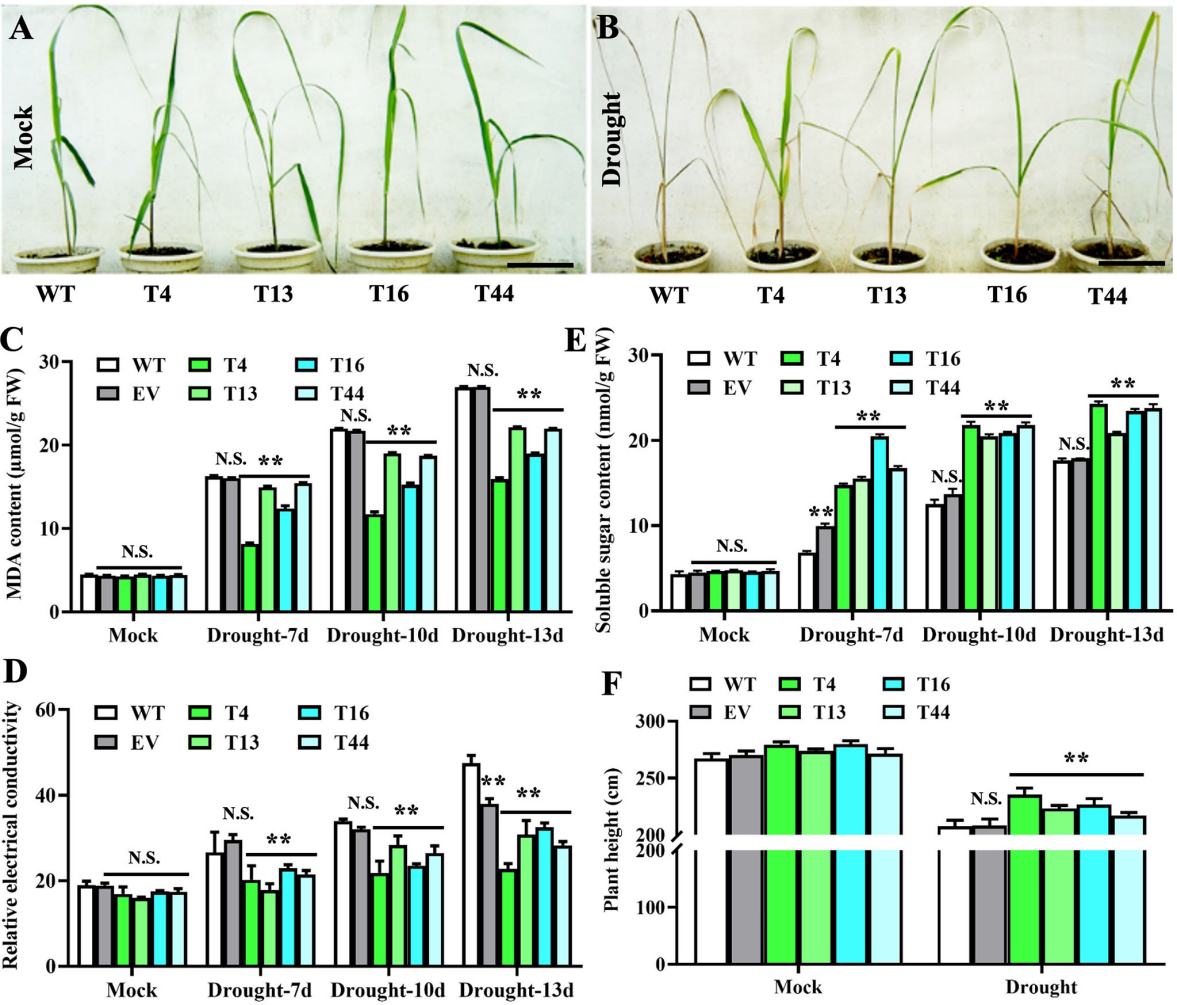
*Prd29A: TaDREB2B* sugarcane positively regulates the drought tolerance without compromising growth in the greenhouse**. (A, B)** Performance of wild type (WT) and *Prd29A: TaDREB2B* transgenic sugarcane seedlings under well-watered **(A)** and severe drought **(B)** conditions. **(C–F)** The determination of malondialdehyde content **(C)**, relative electrical conductivity **(D)**, soluble sugar content **(E)**, and plant height **(F)** in WT, empty vector (EV), and *Prd29A: TaDREB2B* transgenic sugarcane seedlings after drought treatment. EV represents the transgenic sugarcane transformed with an empty vector. The values are the means ± SD; n=6. All statistical analyses were performed using Student’s *t*-test: NS, no significance; **P < 0.01.

To investigate the functions of TaDREB2B in response to drought stress, WT, empty vector control (EV), and four transgenic *Prd29A*:TaDREB2B sugarcane lines (T4, T13, T16, and T44) were subjected to drought (ceasing water for about 15 days), and the seedlings phenotypes were subsequently recorded. No significant growth difference was observed between all sugarcane seedlings at the four-leaf stage under normal growing conditions (**Figure 3A**). However, following drought treatment, transgenic *Prd29A*:TaDREB2B plants show stronger drought tolerance symptoms, including reduced chlorosis and wilting in leaves, than WT (**Figure 3B**). Under normal growth conditions, the MDA content and relative electrical conductivity have no significant differences between *Prd29A*:TaDREB2B and WT sugarcane (**Figures 3C, D**). Drought stress increased the MDA content and relative electrical conductivity in transgenic-, WT, and EV seedlings. However, these stress markers in *Prd29A*:TaDREB2B sugarcane were lower than that in WT and EV (**Figures 3C, D**), indicating that the expression of TaDREB2B under *RD29A* promoter in sugarcane resulted in better cell membrane integrity, and caused minor damage. Additionally, drought stress increased the proline content in *Prd29A*:TaDREB2B and WT sugarcane seedlings. However, the accumulation of proline in *Prd29A*:TaDREB2B plants was obviously higher compared with WT plants (**Figure 3E**), indicating that the expression of TaDREB2B in transgenic sugarcane could enhance the osmotic adjusting ability of cells. In addition, the plant height of WT and *Prd29A*:TaDREB2B transgenic sugarcane was measured at the maturation stage during water deficit. All sugarcane seedlings have no significant difference in plant height under well-watered conditions. However, the plant height of *Prd29A*:TaDREB2B sugarcane lines increased by 4.4%-13.3% than WT under drought stress (**Figure 3F**), suggesting that *Prd29A*:TaDREB2B improved drought tolerance of sugarcane without penalized growth. To comprehensively evaluate the drought resistance of the WT and *TaDREB2B*-transgenic sugarcane, four crucial physiological and biochemical indexes of the transgenic sugarcane were analyzed using membership functions (**Table 1**). The total evaluation value of all transgenic sugarcane lines was calculated. Higher comprehensive evaluation values represent stronger drought-resistance ability. The results showed that the order of drought resistance of the sugarcane lines was as follows: T4> T16> T13 > T44> EV> WT (**Table 1**).

**Table 1:** Membership function values and evaluation index of the drought resistance of TaDREB2B-transgenic sugarcane.

Lines	Membership function value				Syntheticevaluation	Drought resistance
	MDA	Soluble sugar	Relative electrical conductivity	Plant height injury rate		
WT	0.01	0	0.05	0.089	0.037	6
EV	0.109	0.167	0.194	0	0.117	5
T_4_	0.99	0.876	0.804	1	0.918	1
T_13_	0.357	0.549	0.828	0.621	0.589	3
T_16_	0.673	0.7	0.655	0.547	0.644	2
T_44_	0.205	0.881	0.563	0.385	0.508	4

### Prd29A:TaDREB2B improves the survival of sugarcane during drought and post-drought recovery

**Figure 4:**
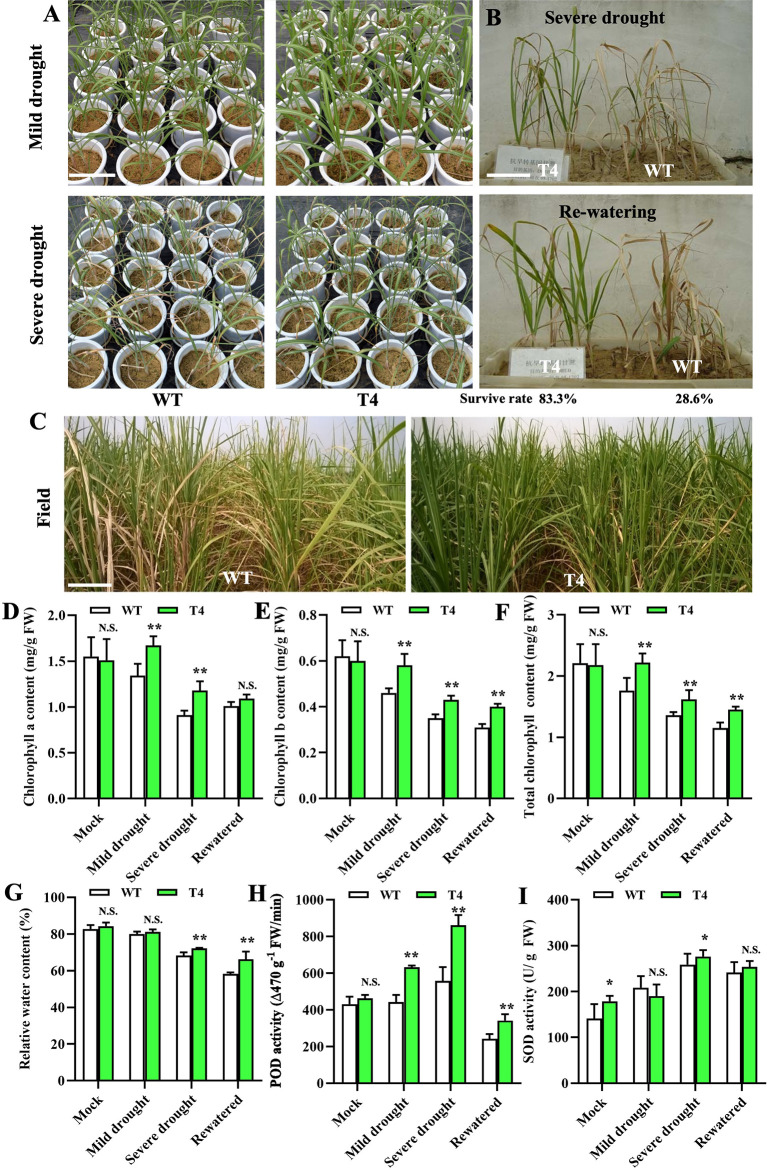
TaDREB2B increases drought tolerance in sugarcane in the field. **(A)** Performance of wild type (WT) and *Prd29A: TaDREB2B* transgenic sugarcane seedlings under mild and severe drought conditions in the greenhouse. **(B)** The survival rate of WT and *Prd29A: TaDREB2B* transgenic seedlings planted in the same nursery pots under severe drought conditions and after re-watered treatment. **(C)** Performance of WT and *Prd29A: TaDREB2B* transgenic sugarcane under the water-limited condition in the field. **(D–I)** The chlorophyll a content **(D)**, chlorophyll b content **(E)**, total chlorophyll content **(F)**, relative water content **(G)**, POD activity **(H)**, and SOD activity **(I)** of WT and *Prd29A: TaDREB2B* transgenic seedlings under different water-limited and re-watered conditions. The values are the means ± SD; n=6. All statistical analyses were performed using Student’s *t*-test: N.S., not significance; *P < 0.05; **P < 0.01.

Based on the physiological characteristics examination and membership function analysis, we further selected the most drought-tolerant *Prd29A:TaDREB2B* transgenic sugarcane line (T4) to evaluate drought-related utility in the field. We performed three analyses: (1) drought resistance experiments of the *Prd29A:TaDREB2B* sugarcane at the seedling stage in the greenhouse and during vigorous growth period in the field; (2) determination of key physiological characteristics of the *Prd29A:TaDREB2B* sugarcane in pots cultivation under rainproof shelter; (3) yield comparisons and examination of agronomic traits in the field with controlled irrigation conditions. We first performed a drought tolerance analysis for the WT and T4 line in the greenhouse. After stopping water for 7 d (mild drought), the WT seedlings displayed distinct growth retardation, but T4 line plants were still thriving. After stopping water for 15 d (severe drought), WT showed evident drought symptoms, such as leaf rolling, while the degree of leaf rolling in the T4 line was notably very slight than WT (**Figure 4A**). We subsequently planted the T4 line and WT seedlings side-by-side in pots. After stopping water for 15 d, WT seedlings at the three-leaf stage exhibited higher water stress symptoms that most leaves were fully curled and withered, while the transgenic T4 line leaves remained green (**Figure 4B**). 3-5 days after rehydration, most WT plants failed to recover and then die, leading to only a 28.6% survival rate, but 83.3% of the T4 plants survived (**Figure 4B**). We further investigated the drought tolerance of the WT and T4 line sugarcane at a vigorous growth period in the field under controlled irrigation conditions. We found that deficit irrigation completely withered the leaves of WT plants while the T4 line showed less severe wilting (**Figure 4C**). The other examination results of a series of physiological characteristics were consistent with these phenotypes. Under normal growth conditions, the chlorophyll contents have no a significant difference between WT and T4 lines, whereas, under drought or re-watering conditions, the contents of chlorophyll a/b and total chlorophyll were significantly higher in T4 line plants than in WT (**Figures 4D–F**). Moreover, after drought stress, the relative water content of T4 line leaves was higher than WT (**Figure 4G**), suggesting that leaves of the T4 sugarcane have a more substantial water retention capacity. Drought stress-induced POD and SOD activity in both the T4 line and WT sugarcane, and the level of POD and SOD activity in the T4 sugarcane was significantly higher compared with WT (**Figures 4H, I**), suggesting that sugarcane plants expressing *TaDREB2B* exhibit higher antioxidant enzyme activity to reduce cell damage.

### Prd29A:TaDREB2B sugarcane exhibits improved agronomic traits during water deficit in the field

**Figure 5:**
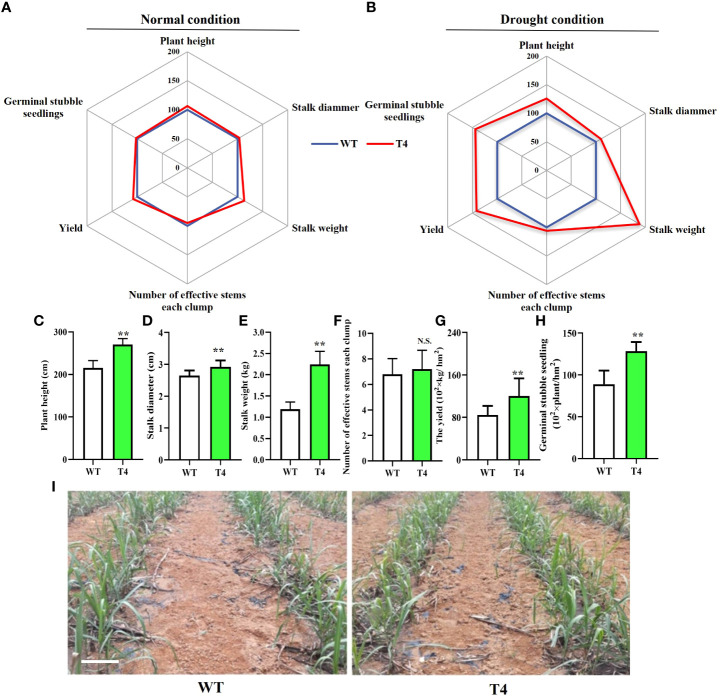
Agronomic traits of wild type (WT) and *Prd29A:TaDREB2B* transgenic (T4) sugarcane under the water-limited conditions in the field. **(A, B)** Agronomic traits of *Prd29A: TaDREB2B* transgenic and WT plants grown in the field under both normal **(A)** and drought **(B)** conditions. Mean values from WT were set at 100% as a reference. Each data point shows a percentage of the mean values. **(C–H)** The plant height **(C)**, stalk diammer **(D)**, stalk weight **(E)**, number of effective stems for each clump **(F)**, the yield **(G)**, and germinal stubble seedlings **(H)** of WT and *Prd29A: TaDREB2B* transgenic sugarcane under water deficit condition. The values are the means ± SD; n=60 plants in **(C–E)**, n=9 clumps in **(F)**, and n=3 in **(G)**. All statistical analyses were performed using Student’s *t*-test: NS, no significance; **P < 0.01. **(I)** Performance of germinal stubble seedlings of WT and *Prd29A: TaDREB2B* transgenic sugarcane after harvest following spring. Bar=20 cm.

The greenhouse and field experiments indicated that the transgenic expression of *TaDREB2B* increases drought tolerance, we subsequently planted all transgenic sugarcane to evaluate field performance for agronomic traits at the maturation stage under normal and drought conditions. Transgenic T4 and WT were not different in several critical agronomic traits, including plant height, stalk diameter, stalk weight, and yield in the field under the normal condition (**Figure 5A**). However, under limited-irrigation conditions, these critical agronomic traits of T4 transgenic sugarcane were visibly superior to that in WT (**Figure 5B**), such as plant height, stalk diameter, and stalk weight (**Figures 5C–E**), leading to the yield of the T4 line significantly increasing by > 40% (**Figure 5G**), while there was no significant difference in the number of effective stems between the T4 line and WT sugarcane (**Figure 5F**). In addition, other *TaDREB2B*-transgenic sugarcane lines also show better agronomic traits than WT and EV sugarcane under water-limited condition in the field (**Supplemental Figure 2**). We also investigated the number of germinal stubble seedlings in the following spring after harvesting WT and T4 line sugarcane plants at the maturation stage under limited-irrigation conditions. We found that the seedlings in T4 line sugarcane were increased by 44.4% more than WT (**Figures 5H, I**). These results indicated that the increase in expression level of *TaDREB2B* could improve the performance of multiple traits and enhance sugarcane yield.

### Prd29A:TaDREB2B sugarcane maintains key quality traits under the limited-irrigation condition in the field

**Figure 6:**
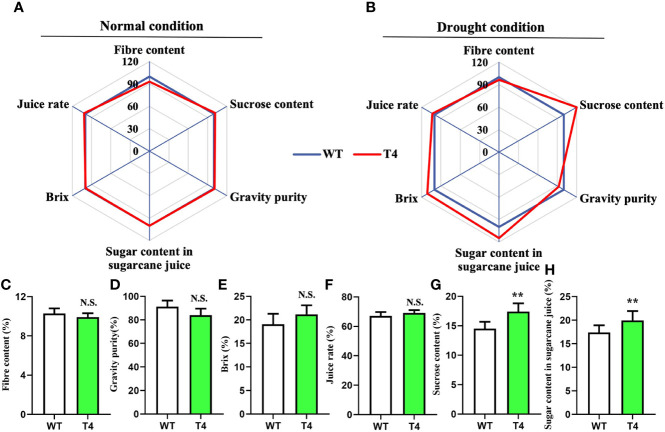
Quality traits of wild type (WT) and *Prd29A:TaDREB2B* transgenic sugarcane during water deficit in the field. **(A, B)** Quality traits of *Prd29A: TaDREB2B* transgenic and WT plants grown in the field under both normal **(A)** and drought **(B)** conditions. Mean values from WT were set at 100% as a reference. Each data point shows a percentage of the mean values. **(C–H)** Fiber content **(C)**, gravity purity **(D)**, Brix **(E)**, juice rate **(F)**, sucrose content **(G)**, and sugar content in sugarcane juice **(H)** of WT and *Prd29A: TaDREB2B* transgenic plants harvested under water-limited conditions. The values are the means ± SD; n=60 plants. All statistical analyses were performed using Student’s *t*-test: NS, no significance; **P < 0.01.

We next monitored several key quality traits in the *Prd29A:TaDREB2B* transgenic sugarcane to access its application potential in sugarcane breeding, including fiber content, sucrose content, gravity purity, juice rate, Brix and sugar content in sugarcane juice, under normal and drought conditions. No difference was observed between WT and T4 line under normal growth conditions (**Figure 6A**). And under the drought condition, the fiber content, gravity purity, Brix, and juice rate of T4 plants and WT have not significant difference (**Figures 6B–F**), while the sucrose content and the sugar content in sugarcane juice of T4 plants significantly higher than WT (**Figures 6G, H**). Taken together, these results suggest that the expression of *TaDREB2B* under the *RD29A* promoter does not penalize major quality traits.

## Discussion

Because of the very limited genetic diversity of modern sugarcane cultivar, this bottleneck severely hindered the genetic improvement of sugarcane in multiple traits such as drought tolerance. In recent years, increasing researchers constantly broadened the genetic background of sugarcane by introducing excellent gene resources from closely related genera, such as *Tripidium*, *Miscanthus*, and *Sclerostachya* (Singh et al., 2011; Wu et al., 2014). For example, *T. arundinaceum* acts as a relative wild species of the genus *Saccharum* and is well known for its excellent drought tolerance, disease resistance, and ratooning ability. In recent decades, researchers attempted to obtain hybrid offspring between sugarcane and *T. arundinaceum* or introduce the favorable genes from *T. arundinaceum* to realize the germplasm innovation of sugarcane (Wu et al., 2014; Huang et al., 2015). A *T. arundinaceum-*specific primer pair AGRP52/53 obtained through genomic *in situ* hybridization was used as a molecular marker to assist breeders to select hybrid progenies of *Saccharum* spp. and *T. arundinaceum* (Yang et al., 2019). The overexpression of a *Glyoxalase III* gene from *T. arundinaceum* promotes the accumulation of chlorophyll, proline, and soluble sugars in transgenic sugarcane upon water deficit stress, and thus enhances drought tolerance in sugarcane (Mohanan et al., 2020). A heat shock protein *HSP70* isolated from *T. arundinaceum* enhanced tolerance to drought and salinity in sugarcane through improving cell membrane thermostability, photosynthetic efficiency, etc (Augustine et al., 2015a). These studies demonstrated that introducing favorable genes from sugarcane relatives provides an effective way to improve stress tolerance in sugarcane. However, these researches on drought tolerance-related genes were performed in growth chambers or greenhouses, there are few studies to demonstrate their application value in the field. Moreover, it is unclear whether these genes affect the yield or quality of sugarcane. We identified a DREB protein TaDREB2B from *T. arundinaceum* and generated transgenic sugarcane plants expressing *TaDREB2B*. Our findings indicate that introducing *Prd29A: TaDREB2B* into the commercial sugarcane cultivar FN95-1702 affords a more remarkable survival rate in the greenhouse and better-growing status in the field when subjected to severe drought (**Figure 4**). In particular, it also leads to higher sugarcane yield and not penalized major quality traits than WT cultivars under drought conditions (**Figure 5**). In addition, sugarcane production costs and planting benefits also depend on the sugarcane ratooning ability, and the cost of ratoon cane is 25%–30% lower than that of new-planted sugarcane, i.e., plant cane (Xu et al., 2021). In our study, *TaDREB2B*-transgenic sugarcane has more germinal stubble seedlings of ratoon cane than WT (**Figure 5**), suggesting that *TaDREB2B*-transgenic sugarcane has more vital ratooning ability. Collectively, our study provides a drought candidate gene *TaDREB2B* which will be a promising biotechnological tool for sugarcane drought-resistant breeding applications in the future.

Numerous studies revealed that constitutive overexpression of stress-related transcription factors usually enhances plant survival under different stress conditions (Feng et al., 2020; Wang et al., 2020; Hu et al., 2021a; Hu et al., 2021b). However, the overexpression of *CBF/DREB* genes often leads to decreased application value in crop improvement due to negative phenotypes in transgenic plants, such as growth retardation, delayed flowering, and reduced biomass (Hsieh et al., 2002; Ito et al., 2006; Morran et al., 2011). For example, *TaDREB3-*overexpressing barley enhanced frost tolerance at the vegetative stage but led to stunted growth and delays in flowering (Kovalchuk et al., 2013). Overexpression of *LbDREB6* significantly improved drought tolerance in poplar but inhibited growth (Yang et al., 2020a). Similarly, *AtDREB1A-*overexpressed soybeans showed dwarfism and delayed flowering under normal conditions (Suo et al., 2016). Because of this negative influence of constitutive over-expressed promoters, many studies used stress-inducible promoters to replace them (Yang et al., 2020b). *RD29A* in *Arabidopsis* is a well-known drought-responsive gene, and thus its promoter has been attempted to use as a drought-inducible promoter in transgenic biotechnology (Kasuga, 2004; Wei et al., 2016). For example, expression of *DREB1A* controlled by the stress-inducible *RD29A* promoter instead of the CaMV 35S promoter minimizes the adverse effects on *Arabidopsis* growth (Kasuga et al., 1999). The direct drive of *CBF* by the *RD29A* promoter in potato induces low background expression under non-stressful conditions, leading to distinctly increased freezing tolerance and no negative impact on key agronomical traits (Pino et al., 2007). Based on the above findings, we also selected the *RD29A* promoter to drive the expression of *TaDREB2B* in this study. Our previous study revealed that GFP expression driven by the *RD29A* promoter could be significantly induced by PEG stress in sugarcane callus (Wu et al., 2008). The resulting construct (*Prd29A: TaDREB2B*) was then introduced into a commercial sugarcane cultivar FN95-1702. The expression of *TaDREB2B* controlled by the *RD29A* promoter can be distinctly induced in a mild drought and slightly increased in severe drought stress (**Figure 2**). However, after treatment at rehydration, this induction of *TaDREB2B* expression was subsequently decreased (**Figure 2**), suggesting that the use of *RD29A* promoter could avoid excessive and prolonged drought activation response to ensure limited metabolic energy be more used more for normal growth of the plant. Transgenic T4 line carrying *Prd29A: TaDREB2B* exhibited more remarkable survival under severe drought (**Figure 4**), but no significant reduction in agronomic traits and major quality traits under normal conditions (**Figures 5**, **6**), which unlike adverse effects resulting from CaMV 35S promoter. Moreover, the *TaDREB2B*-transgenic sugarcane plants using *RD29A* promote had more substantial drought tolerance and had higher yield potential. Therefore, in the future, it will be essential to consider the stress-inducible promoter, design and apply these promoters in sugarcane breeding to redistribute resources, and balance stress tolerance and sugarcane yield.

## Conclusions

This study revealed that the transformation of the *TaDREB2B* gene under the control of the drought-responsive *RD29A* promotes improved the tolerance of the key commercial sugarcane cultivar FN95-1702 to drought stress to a great extent. *Prd29A: TaDREB2B* transgenic sugarcane exhibits enhanced yield and uncompromising major quality traits during water deficit in the field; thus, *Prd29A: TaDREB2B* will be a useful biotechnological tool for sugarcane drought-resistance breeding in the future.

## Data availability statement

The original contributions presented in the study are included in the article/**Supplementary Material.** Further inquiries can be directed to the corresponding author.

## Author contributions

MQZ conceived and designed the project and the experiments. SHX and YW performed most of the assays and wrote the manuscript draft; SQX and HTJ helped perform the drought treatment experiments and data analysis. MQZ, WY, and QH revised it. All the authors discussed the results and the conception of the article.

## Funding

This work was supported by funding from China Agricultural Research Systems Funded by MOF and MARA (CARS170109), Guangxi Natural Science Foundation (2021JJB130425), Scientific Research and Development Fund of College of Agriculture, Guangxi University (EE101711).

## Conflict of interest

The authors declare that the research was conducted in the absence of any commercial or financial relationships that could be construed as a potential conflict of interest.

## Publisher’s note

All claims expressed in this article are solely those of the authors and do not necessarily represent those of their affiliated organizations, or those of the publisher, the editors and the reviewers. Any product that may be evaluated in this article, or claim that may be made by its manufacturer, is not guaranteed or endorsed by the publisher.

## References

[B1] Agarwal P. Gupta K. Lopato S. Agarwal P. (2017). Dehydration responsive element binding transcription factors and their applications for the engineering of stress tolerance. J. Exp. Bot. 68 (9), 2135–2148. doi: 10.1093/jxb/erx118 28419345

[B2] Arencibia A. D. Carmona E. R. Cornide M. T. Castiglione S. O’Relly J. Chinea A.. (1999). Somaclonal variation in insect-resistant transgenic sugarcane (Saccharum hybrid) plants produced by cell electroporation. Transgenic Res. 8 (5), 349–360. doi: 10.1023/A:1008900230144

[B3] Augustine S. Narayan J. Syamaladevi D. Appunu C. Chakravarthi M. Ravichandran V.. (2015a). Erianthus arundinaceus HSP70 (EaHSP70) overexpression increases drought and salinity tolerance in sugarcane (Saccharum spp. hybrid). Plant Sci. an Int. J. Exp. Plant Biol. 232, 23–34. doi: 10.1016/j.plantsci.2014.12.012 25617320

[B4] Augustine S. M. Syamaladevi D. P. Premachandran M. N. Ravichandran V. Subramonian N. (2015b). Physiological and molecular insights to drought responsiveness in erianthus spp. Sugar Tech 17 (2), 121–129. doi: 10.1007/s12355-014-0312-7

[B5] Feng X. Liu W. Dai H. Qiu Y. Zhang G. Chen Z.. (2020). HvHOX9, a novel homeobox leucine zipper transcription factor, positively regulates aluminum tolerance in Tibetan wild barley. J. Exp. Bot. 71 (19), 6057–6073. doi: 10.1093/jxb/eraa290 32588054

[B6] Garcia Tavares R. Lakshmanan P. Peiter E. O'Connell A. Caldana C. Vicentini R.. (2018). ScGAI is a key regulator of culm development in sugarcane. J. Exp. Bot. 69 (16), 3823–3837. doi: 10.1093/jxb/ery180 29767776 PMC6054169

[B7] Gilbert R. A. Glynn N. C. Comstock J. C. Davis M. J. (2009). Agronomic performance and genetic characterization of sugarcane transformed for resistance to sugarcane yellow leaf virus. Field Crops Res. 111 (1-2), 39–46. doi: 10.1016/j.fcr.2008.10.009

[B8] Hsieh T. Lee J. Yang P. Chiu L. Charng Y. Wang Y.. (2002). Heterology expression of the *Arabidopsis* c-repeat/dehydration response element binding factor 1 gene confers elevated tolerance to chilling and oxidative stresses in transgenic tomato. Plant Physiol. 129 (3), 1086–1094. doi: 10.1104/pp.003442 12114563 PMC166503

[B9] Huang Y. Luo L. Hu X. Yu F. Yang Y. Deng Z.. (2017). Erianthus arundinaceusCharacterization, genomic organization, abundance, and chromosomal distribution of Ty1-copia retrotransposons in. Front. Plant Sci. 8. doi: 10.3389/fpls.2017.00924 PMC5461294 28638390

[B10] Huang Y. Wu J. Wang P. Lin Y. Fu C. Deng Z.. (2015). Characterization of chromosome inheritance of the intergeneric BC2 and BC3 progeny between saccharum spp. and erianthus arundinaceus. PloS One 10 (7), e0133722. doi: 10.1371/journal.pone.0133722 26196281 PMC4510360

[B11] Hu Q. Ao C. Wang X. Wu Y. Du X. (2021a). GhWRKY1-like, a WRKY transcription factor, mediates drought tolerance in *Arabidopsis via* modulating ABA biosynthesis. BMC Plant Biol. 21 (1), 458. doi: 10.1186/s12870-021-03238-5 34625048 PMC8501554

[B12] Hu Q. Xiao S. Wang X. Ao C. Zhang X. Zhu L. (2021b). GhWRKY1-like enhances cotton resistance to verticillium dahliae *via* an increase in defense-induced lignification and s monolignol content. Plant Sci. an Int. J. Exp. Plant Biol. 305, 110833. doi: 10.1016/j.plantsci.2021.110833 33691967

[B13] Ito Y. Katsura K. Maruyama K. Taji T. Kobayashi M. Seki M.. (2006). Functional analysis of rice DREB1/CBF-type transcription factors involved in cold-responsive gene expression in transgenic rice. Plant Cell Physiol. 47 (1), 141–153. doi: 10.1093/pcp/pci230 16284406

[B14] Kasuga M. (2004). A combination of the *Arabidopsis* DREB1A gene and stress-inducible rd29A promoter improved drought- and low-temperature stress tolerance in tobacco by gene transfer. Plant Cell Physiol. 45 (3), 346–350. doi: 10.1093/pcp/pch037 15047884

[B15] Kasuga M. Liu Q. Miura S. Yamaguchi-Shinozaki K. Shinozaki K. (1999). Improving plant drought, salt, and freezing tolerance by gene transfer of a single stress-inducible transcription factor. Nat. Biotechnol. 17 (3), 287–291. doi: 10.1038/7036 10096298

[B16] Kovalchuk N. Jia W. Eini O. Morran S. Pyvovarenko T. Fletcher S.. (2013). Optimization of TaDREB3 gene expression in transgenic barley using cold-inducible promoters. Plant Biotechnol. J. 11 (6), 659–670. doi: 10.1111/pbi.12056 23495849

[B17] Kudo M. Kidokoro S. Yoshida T. Mizoi J. Todaka D. Fernie A.. (2017). Double overexpression of DREB and PIF transcription factors improves drought stress tolerance and cell elongation in transgenic plants. Plant Biotechnol. J. 15 (4), 458–471. doi: 10.1111/pbi.12644 27683092 PMC5362684

[B18] Liao X. Guo X. Wang Q. Wang Y. Zhao D. Yao L.. (2017). Overexpression of MsDREB6.2 results in cytokinin-deficient developmental phenotypes and enhances drought tolerance in transgenic apple plants. Plant J. 89 (3), 510–526. doi: 10.1111/tpj.13401 27754576

[B19] Li C. Nong Q. Solanki M. K. Liang Q. Xie J. Liu X.. (2016). Differential expression profiles and pathways of genes in sugarcane leaf at elongation stage in response to drought stress. Sci. Rep. 6, 25698. doi: 10.1038/srep25698 27170459 PMC4864372

[B20] Lloyd Evans D. Joshi S. V. Wang J. (2019). Whole chloroplast genome and gene locus phylogenies reveal the taxonomic placement and relationship of tripidium (Panicoideae: Andropogoneae) to sugarcane. BMC Evol. Biol. 19 (1), 33. doi: 10.1186/s12862-019-1356-9 30683070 PMC6347779

[B21] Luo X. Li C. He X. Zhang X. Zhu L. (2020). ABA signaling is negatively regulated by GbWRKY1 through JAZ1 and ABI1 to affect salt and drought tolerance. Plant Cell Rep. 39 (2), 181–194. doi: 10.1007/s00299-019-02480-4 31713664

[B22] Mallikarjuna G. Mallikarjuna K. Reddy M. Kaul T. (2011). Expression of OsDREB2A transcription factor confers enhanced dehydration and salt stress tolerance in rice (Oryza sativa l.). Biotechnol. Lett. 33 (8), 1689–1697. doi: 10.1007/s10529-011-0620-x 21528404

[B23] Manoj V. M. Anunanthini P. Swathik P. C. Dharshini S. Ashwin Narayan J. Manickavasagam M.. (2019). Comparative analysis of glyoxalase pathway genes in erianthus arundinaceus and commercial sugarcane hybrid under salinity and drought conditions. BMC Genomics 19 (Suppl 9), 986. doi: 10.1186/s12864-018-5349-7 30999852 PMC7402403

[B24] Mohanan M. V. Pushpanathan A. Sasikumar S. P. T. Selvarajan D. Jayanarayanan A. N. AK R.. (2020). Ectopic expression of DJ-1/PfpI domain containing erianthus arundinaceus glyoxalase III (EaGly III) enhances drought tolerance in sugarcane. Plant Cell Rep. 39 (11), 1581–1594. doi: 10.1007/s00299-020-02585-1 32876807

[B25] Morran S. Eini O. Pyvovarenko T. Parent B. Singh R. Ismagul A.. (2011). Improvement of stress tolerance of wheat and barley by modulation of expression of DREB/CBF factors. Plant Biotechnol. J. 9 (2), 230–249. doi: 10.1111/j.1467-7652.2010.00547.x 20642740

[B26] Nakashima K. Tran L. Van Nguyen D. Fujita M. Maruyama K. Todaka D.. (2007). Functional analysis of a NAC-type transcription factor OsNAC6 involved in abiotic and biotic stress-responsive gene expression in rice. Plant J. Cell Mol. Biol. 51 (4), 617–630. doi: 10.1111/j.1365-313X.2007.03168.x 17587305

[B27] Pino M. Skinner J. Park E. Jeknić Z. Hayes P. Thomashow M.. (2007). Use of a stress inducible promoter to drive ectopic AtCBF expression improves potato freezing tolerance while minimizing negative effects on tuber yield. Plant Biotechnol. J. 5 (5), 591–604. doi: 10.1111/j.1467-7652.2007.00269.x 17559519

[B28] Que Y. Xu L. Lin J. Luo J. Xu J. Zheng J.. (2012). cDNA-SRAP and its application in differential gene expression analysis: a case study in erianthus arundinaceum. J. BioMed. Biotechnol. 2012, 390107. doi: 10.1155/2012/390107 22778549 PMC3388624

[B29] Ram B. Sreenivasan T. V. Sahi B. K. Singh N. (2001). Introgression of low temperature tolerance and red rot resistance from erianthus in sugarcane. Euphytica 122 (1), 145–153. doi: 10.1023/A:1012626805467

[B30] Rocha F. Papini-Terzi F. Nishiyama M. Vêncio R. Vicentini R. Duarte R.. (2007). Signal transduction-related responses to phytohormones and environmental challenges in sugarcane. BMC Genomics 8, 71. doi: 10.1186/1471-2164-8-71 17355627 PMC1852312

[B31] Sakuma Y. Maruyama K. Qin F. Osakabe Y. Shinozaki K. Yamaguchi-Shinozaki K. (2006). Dual function of an *Arabidopsis* transcription factor DREB2A in water-stress-responsive and heat-stress-responsive gene expression. Proc. Natl. Acad. Sci. United States America 103 (49), 18822–18827. doi: 10.1073/pnas.0605639103 PMC1693746 17030801

[B32] Selvaraj M. G. Jan A. Ishizaki T. Valencia M. Dedicova B. Maruyama K.. (2020). Expression of the CCCH-tandem zinc finger protein gene OsTZF5 under a stress-inducible promoter mitigates the effect of drought stress on rice grain yield under field conditions. Plant Biotechnol. J. 18 (8), 1711–1721. doi: 10.1111/pbi.13334 31930666 PMC7336284

[B33] Singh R. K. Singh R. B. Singh S. P. Sharma M. L. (2011). Identification of sugarcane microsatellites associated to sugar content in sugarcane and transferability to other cereal genomes. Euphytica 182 (3), 335–354. doi: 10.1007/s10681-011-0484-0

[B34] Suo H. Lü J. Ma Q. Yang C. Y. Zhang X. X. Meng X.. (2016). The AtDREB1A transcription factor up-regulates expression of a vernalization pathway gene, GmVRN1-like, delaying flowering in soybean. Acta Physiologiae Plantarum 38 (6), 137. doi: 10.1007/s11738-016-2136-4

[B35] Wang D. Jiang C. Liu W. Wang Y. (2020). The WRKY53 transcription factor enhances stilbene synthesis and disease resistance by interacting with MYB14 and MYB15 in Chinese wild grape. J. Exp. Bot. 71 (10), 3211–3226. doi: 10.1093/jxb/eraa097 32080737

[B36] Wei T. Deng K. Liu D. Gao Y. Liu Y. Yang M.. (2016). Ectopic expression of DREB transcription factor, AtDREB1A, confers tolerance to drought in transgenic salvia miltiorrhiza. Plant Cell Physiol. 57 (8), 1593–1609. doi: 10.1093/pcp/pcw084 27485523

[B37] Wu J. Huang Y. Lin Y. Fu C. Liu S. Deng Z.. (2014). Unexpected inheritance pattern of erianthus arundinaceus chromosomes in the intergeneric progeny between saccharum spp. and erianthus arundinaceus. PloS One 9 (10), e110390. doi: 10.1371/journal.pone.0110390 25310831 PMC4195721

[B38] Wu Y. Zhou H. Que Y. X. Chen R. K. Zhang M. Q. (2008). Cloning and identification of promoter Prd29A and its application in sugarcane drought resistance. Sugar Tech 10 (1), 36–41. doi: 10.1007/s12355-008-0006-0

[B39] Xianjun P. Xingyong M. Weihong F. Man S. Liqin C. Alam I.. (2011). Improved drought and salt tolerance of *Arabidopsis* thaliana by transgenic expression of a novel DREB gene from leymus chinensis. Plant Cell Rep. 30 (8), 1493–1502. doi: 10.1007/s00299-011-1058-2 21509473

[B40] Xiao S. Hu Q. Zhang X. Si H. Liu S. Chen L.. (2021). Orchestration of plant development and defense by indirect crosstalk of salicylic acid and brassinosteorid signaling *via* transcription factor GhTINY2. J. Exp. Bot. 72 (13), 4721–4743. doi: 10.1093/jxb/erab186 33928361

[B41] Xie Z. Nolan T. Jiang H. Tang B. Zhang M. Li Z.. (2019). The AP2/ERF transcription factor TINY modulates brassinosteroid-regulated plant growth and drought responses in *Arabidopsis* . Plant Cell 31 (8), 1788–1806. doi: 10.1105/tpc.18.00918 31126980 PMC6713308

[B42] Xin H. Luo X. Wang T. Liu S. Zhu L. (2020). GhHB12 negatively regulates abiotic stress tolerance in *Arabidopsis* and cotton. Environ. Exp. Bot. 176, 104087. doi: 10.1016/j.envexpbot.2020.104087

[B43] Xiong S. Wang Y. Chen Y. Gao M. Zhao Y. Wu L. (2022). Effects of drought stress and rehydration on physiological and biochemical properties of four oak species in China. Plants (Basel Switzerland) 11, (5). doi: 10.3390/plants11050679 PMC8912384 35270149

[B44] Xu F. Wang Z. Lu G. Zeng R. Que Y. (2021). Sugarcane ratooning ability: Research status, shortcomings, and prospects. Biology 10, (10). doi: 10.3390/biology10101052 PMC8533141 34681151

[B45] Xu S. Wang J. Shang H. Huang Y. Yao W. Chen B.. (2018). Transcriptomic characterization and potential marker development of contrasting sugarcane cultivars. Sci. Rep. 8 (1), 1683. doi: 10.1038/s41598-018-19832-x 29374206 PMC5785991

[B46] Yang Y. Al-Baidhani H. Harris J. Riboni M. Li Y. Mazonka I.. (2020b). DREB/CBF expression in wheat and barley using the stress-inducible promoters of HD-zip I genes: impact on plant development, stress tolerance and yield. Plant Biotechnol. J. 18 (3), 829–844. doi: 10.1111/pbi.13252 31487424 PMC7004899

[B47] Yang J. Wang H. Zhao S. Liu X. Zhang X. Wu W.. (2020a). LbDREB6Overexpression levels of differentially affect growth, drought, and disease tolerance in poplar. Front. Plant Sci. 11. doi: 10.3389/fpls.2020.528550 PMC7693672 33304356

[B48] Yang S. Zeng K. Chen K. Wu J. Wang Q. Li X.. (2019). Chromosome transmission in BC progenies of intergeneric hybrids between saccharum spp. and erianthus arundinaceus (Retz.) jeswiet. Sci. Rep. 9 (1), 2528. doi: 10.1038/s41598-019-38710-8 30792411 PMC6385618

[B49] Yao W. Ruan M. Qin L. Yang C. Chen R. Chen B.. (2017). Sugarcane mosaic VirusField performance of transgenic sugarcane lines resistant to. Front. Plant Sci. 8. doi: 10.3389/fpls.2017.00104 PMC5296345 28228765

[B50] Younis A. Siddique M. Kim C. Lim K. (2014). RNA Interference (RNAi) induced gene silencing: A promising approach of Hi-tech plant breeding. Int. J. Biol. Sci. 10 (10), 1150–1158. doi: 10.7150/ijbs.10452 25332689 PMC4202031

[B51] Yu F. Chai J. Li X. Yu Z. Yang R. Ding X.. (2021). Tripidium arundinaceumChromosomal characterization of revealed by oligo-FISH. Int. J. Mol. Sci. 22 (16), 8539. doi: 10.3390/ijms22168539 34445245 PMC8395171

[B52] Yu F. Zhao X. Chai J. Ding X. Li X. Huang Y.. (2022). Chromosome-specific painting unveils chromosomal fusions and distinct allopolyploid species in the Saccharum complex. New Phytol. 233 (4), 1953–1965. doi: 10.1111/nph.17905 34874076

[B53] Zhou Y. Chen M. Guo J. Wang Y. Min D. Jiang Q.. (2020). Overexpression of soybean DREB1 enhances drought stress tolerance of transgenic wheat in the field. J. Exp. Bot. 71 (6), 1842–1857. doi: 10.1093/jxb/erz569 31875914 PMC7242075

