# Supplementary material for: Field evaluation of TaDREB2B-ectopic expression sugarcane (Saccharum spp. hybrid) for drought tolerance
Source: Front Plant Sci. 2022 Nov 1;13:963377. doi: 10.3389/fpls.2022.963377 (PMC9664057; doi:10.3389/fpls.2022.963377)
Supplement: Supplementary file 1 [file DataSheet_1.pdf]

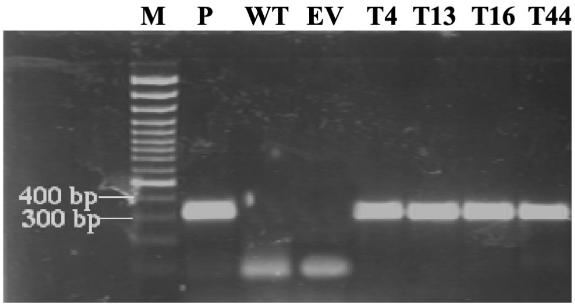

Supplemental Figure 1. RT-PCR analysis of *TaDREB2B* expression in transgenic sugarcane.

M: DL 2000 marker; P: Plasmid DNA; WT: wild type; EV: empty vector; T4-T44: *TaDREB2B*-transgenic sugarcane.

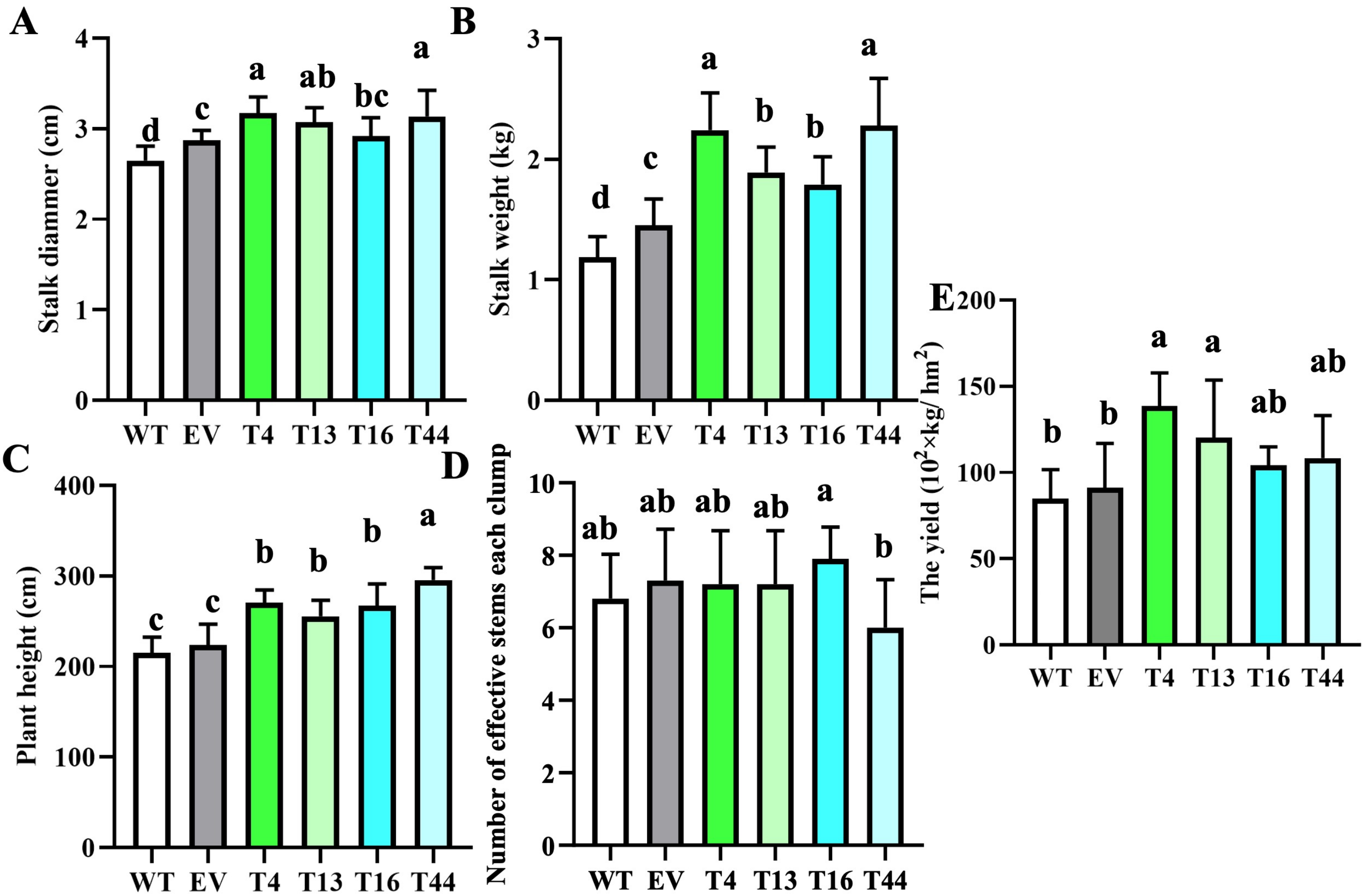

Supplemental Figure 2. Agronomic traits of WT and *TaDREB2B* transgenic lines under water-limited condition in the field.

(A-E) The major agronomic traits determined, including stalk diammer (A), stalk weight (B), plant height (C), number of deffective stems each clump (D) and the yield (E). The values are the means ± SD; n=60 plants in (A-C), n=9 clumps in (D) and n=3 in (E). Different letters indicate significant differences as determined using ANOVA and LSD multiple comparisons ($P<0.05$).
